# Knockout of *EGFL6* by CRISPR/Cas9 Mediated Inhibition of Tumor Angiogenesis in Ovarian Cancer

**DOI:** 10.3389/fonc.2020.01451

**Published:** 2020-08-25

**Authors:** Wenhui Zhu, Chunyan Liu, Tongyi Lu, Yinmei Zhang, Simin Zhang, Qi Chen, Ning Deng

**Affiliations:** ^1^Guangdong Province Engineering Research Center for Antibody Drug and Immunoassay, Department of Biology, Jinan University, Guangzhou, China; ^2^Department of Pharmacology, Toxicology and Therapeutics, University of Kansas Medical Center, Kansas City, KS, United States

**Keywords:** CRISPR/Cas9, knockout, ovarian cancer, EGFL6, angiogenesis

## Abstract

Tumor angiogenesis plays an important role in the progression and metastasis of ovarian cancer. EGFL6 protein is highly expressed in ovarian cancer and has been proposed to play an important role in promoting tumor angiogenesis. Here, a CRISPR/Cas9 system was used to knockout the *EGFL6* gene in the ovarian cancer cell line SKOV3, using specific guide RNA targeting the exons of *EGFL6*. The knockout of *EGFL6* markedly inhibited the proliferation, migration, and invasion of SKOV3 cells, as well as promoted apoptosis of tumor cells. In the nude mouse model of ovarian cancer, knockout of *EGFL6* remarkably inhibited tumor growth and angiogenesis. The transcript profile assays detected 4,220 differentially expressed genes in the knockout cells, including 87 genes that were correlated to proliferation, migration, invasion, and angiogenesis. Moreover, Western blotting confirmed that *EGFL6* knockout downregulated the FGF-2/PDGFB signaling pathway. Thus, the results of this study indicated that *EGFL6* could regulate cell proliferation, migration, and angiogenesis in ovarian cancer cells by regulating the FGF-2/PDGFB signaling pathway.

## Introduction

Ovarian cancer is a kind of malignant tumor that poses serious threat to women's health and is one of the most lethal gynecological tumors (1, 2). Patients with ovarian cancer are often asymptomatic in the early stages and, therefore, remain undiagnosed until at advanced stages of the disease (3). Ovarian cancer exhibits heterogeneity and includes several subtypes, making its diagnosis difficult and management complex (4). Ovarian cancer is often susceptible to traditional platinum-chemotherapy drugs, albeit with a high recurrence rate and high chemotherapy resistance; it has the highest morbidity rate among gynecological cancers (5).

Tumor angiogenesis plays an important role in the development and metastasis of tumors (1, 6). The angiogenic factors of ovarian cancer can be targeted for inhibiting tumor growth and metastasis. Treatment with bevacizumab has been successful previously, as it inhibits tumor angiogenesis and metastasis (7, 8). During the progression of ovarian cancer, tumors could promote angiogenesis by secreting angiogenic factors such as vascular endothelial growth factor (VEGF), basic fibroblast growth factor (bFGF), and platelet-derived growth factor (PDGF), thereby promoting the neoformation of tumor blood vessels and, in turn, metastasis of ovarian cancer (9–11). Angiogenesis and its related genes have become a hotspot for research on targeted cancer treatment in recent years.

Epidermal growth factor-like domain multiple 6 (EGFL6), belonging to the epidermal growth factor-like protein family (EGFL), was first discovered by Yeung (12). EGFL6 contains an N-terminal signal peptide domain, an integrin binding site, multiple EGF domains, and a C-terminal MAM domain (13). EGFL6 is almost undetectable in normal human tissues but highly expressed in embryos and most tumor tissues (14–16). The murine EGFL6 promotes the migration of porcine vascular endothelial cells (SVEC) through the activation of the ERK signaling pathway and its downstream effector molecules, resulting in angiogenesis (17–20). Bai et al. (21) found that EGFL6 promoted the phosphorylation of ERK, stimulated asymmetric division of ovarian cancer stem cells, and promoted ovarian cancer growth as well as metastasis. In addition, Noh et al. (22) found that EGFL6 was regulated by the transcriptional factor Twist and promoted ovarian cancer angiogenesis by regulating integrin/Tie2/AKT (22). An et al. (23) found that EGFL6 not only is associated with cancer cell proliferation, migration, invasion, and angiogenesis, but also promotes epithelial-to-mesenchymal transition (EMT) and tumor stem cell characteristics in breast cancer. Considering the multiple mechanisms proposed in different cancers, the role of EGFL6 in tumor angiogenesis and progression of ovarian cancer requires further investigation.

The CRISPR/cas9 system comprises a single-strand guide RNA (sgRNA) and a Cas9 protein with endonuclease activity (23). The sgRNA guides the Cas9 protein to cleave the target DNA molecule and form a DNA double-strand break, causing an INDEL effect when it is repaired via non-homologous end joining (NHEJ), thereby resulting in a frame shift mutation of the gene and its knockout (24, 25). CRISPR/Cas9 technology is widely used in the study of gene function and may present new prospects in therapeutics.

In this study, the *EGFL6* was knocked out by CRISPR/Cas9 in an ovarian cancer cell line, using a specific guide RNA (gRNA) designed to target the exons of *EGFL6*. Further, the role of EGFL6 in the proliferation and migration of cells, tumor growth, and angiogenesis in ovarian cancer was assayed. Additionally, the relationship of EGFL6 with the FGF-2 and PDGFB signaling pathways was investigated. However, the molecular mechanism of EGFL6 function in the tumorigenesis of ovarian cancer needs further investigation.

## Materials and Methods

### Tissue Samples of Ovarian Cancer

Four malignant and one normal ovary tissue samples were obtained from the Third Affiliated Hospital of Sun Yat-Sen University (Guangzhou, China).

### Design of sgRNAs for the EGFL6 Gene

The sgRNA was designed according to exon 2 of the human EGFL6 gene and predicted by using an online tool developed by Prof. Zhang (http://crispr.mit.edu/). The gRNA sequences used were sgRNA1 TTAGCATCGGCACGTCAGCC; sgRNA2 GCTGCTACGGCTGGAGAAGA. The non-targeting control gRNA was designed according to the GeCKO v2 library (26). The negative control consisted of the non-targeting control gRNA: AAAGAAAGAGGAATAGTAGC. The gRNA was inserted into lentiCRISPR V2 vectors (Addgene). Double-stranded nucleotide sequences with *BsmBI* restriction sites were synthesized.

### Construction of the Cas9-sgRNA Expression Plasmid

The double-stranded guide sequence oligonucleotides were ligated into lentiCRISPRv2 linearized by *BsmBI* restriction enzyme using T4 DNA ligase and the plasmid was transformed into competent cells of *Escherichia coli*. The plasmid DNA was purified using HiPure Gel Pure Micro Kit (Magen, China, Cat: D2110-02). Colony PCR was performed to verify successful insertion of the guide sequence, using the sequencing primer 5′-GGCCTATTTCCCATGATTCC-3′. Positive clones were selected, and the sequences were verified.

### Cell Culture

Ovarian cancer cell lines (TOV21G, CAOV3, ES2, A2780, SKOV3, COV362, OV90, and COV504), human umbilical vein endothelial cells (HUVECs), and human embryonic kidney (HEK) 293T cells were purchased from the American Type Culture Collection (ATCC, Rockville, MD, USA). All cells were maintained in DMEM medium supplemented with 10% fetal bovine serum (FBS) (BI) and 1% penicillin/streptomycin solution (Gibco). All cell lines were cultured at 37°C under a humidified atmosphere of 5% CO_2_.

### Cloning and Screening of Ovarian Cancer Cells With EGFL6 Gene Knockout

The lentiCRISPRv2 recombinant plasmid along with plasmids of psPAX2 and pMD2G were co-transfected into HEK293T by ViaFect™ Transfection Reagent (Promega, USA, Cat: E4982). Lentivirus was harvested at 48 h. The 2-ml lentivirus solution with 8 μg/ml polybrene (Solarbio, Cat: H8761) was slowly added to SKOV3 cells cultured in a 6-well plate. The medium was changed to complete medium after 24 h and the cells were selectively cultured using 2 μg/ml puromycin (Sigma, USA).

After puromycin selective cultivation, the selected SKOV3 cells were cloned using limited dilution in 96-well plates. The single cell clones were transferred to 24-well plates and cultured for 2 weeks. The cells were harvested and the genomic DNA was extracted using a DNA Extraction Kit (TSINGKE Biological Technology, China, Cat: TSP201-200). The mutation sites were identified by colony PCR using EGFL6 primers (Table 1) and sequencing analysis. The EGFL6 knockout was verified by measurement of protein levels using Western blot.

**Table 1:** PCR primers for amplifying EGFL6 gene fragments.

**Gene names**	**Forward primer (5^**′**^-3^**′**^)** **Reverse primer (5^**′**^-3^**′**^)**
EGFL6-Exon2-Forward primer	AACTGGATAGTTGCTGTGCGT
EGFL6-Exon2-Reverse primer	TCCACTGGCAATTTTGTGGC

### Western Blotting

The Western blotting experiment procedure was performed as described previously (23). The rabbit anti-human EGFL6, PDGFB polyclonal antibody, rabbit anti-human VEGFA, FGF-2, integrin beta 1 monoclonal antibody (Abcam, 1:1,000, Cat: ab140079, ab23914, ab52917, ab92337, ab183666); rabbit anti-t/p-Akt, anti-t/p-Erk, mTOR, ICAM-1, E-Cadherin, N-Cadherin, Caspase-8, Caspase-3, Bcl-2, Bax (Cell Signaling Technology, 1:1,000, Cat: 4691S, 4060S, 4695S, 4370S, 2983S, 4915S, 3195S, 13116S, 9496S, 14220S, 4223S, 5023S); rabbit anti-human Vimentin polyclonal antibody (proteintech^TM^, Cat: 1:4,000, 10366-1-AP); and rabbit anti-human MMP9 and MMP2 (proteintech^TM^, 1:1,000, Cat: 10375-2-AP, 10373-2-AP) polyclonal antibody were used as primary antibodies. The GAPDH monoclonal antibody (Cell Signaling Technology, 1:1,000, Cat: 5174S) was the control. The secondary antibody used was the HRP-conjugated goat anti-rabbit IgG (Cell Signaling Technology, 1:5,000, Cat: 7074S). The immunoreactive proteins were detected using Immobilon^TM^ Western Chemiluminescent HRP Substrate (Millipore, USA) and a chemiluminescence imaging system (Tanon, China).

### Re-expression of EGFL6 in *EGFL6* Knockout Ovarian Cancer Cells

EGFL6 gene primers were designed (Table 2) and synthesized (TSINGKE Biological Technology, China). The EGFL6 gene was amplified by PCR using a template of the pAAV-GFP-EGFL6 plasmid. The PCR products were inserted into the pCDH513b vector (Shenzhen City Baozhu Biological Technology Co Ltd, China, Cat: zt145) to construct the expression vector pCDH513b-EGFL6 according to the Trelief™ SoSoo Cloning Kit instructions (TSINGKE Biological Technology, China, Cat: TSV-S1).

**Table 2:** PCR primers for amplifying EGFL6 gene.

**Gene names**	**Forward primer (5^**′**^-3^**′**^)** **Reverse primer (5^**′**^-3^**′**^)**
EGFL6-Sosoo-Forward primer	CTGTTTTGACCTCCATAGAAGATTCTAGAATGCCTCTGCCCTGGAGCCTTG
EGFL6-Sosoo-Reverse primer	CTTGCGGCCGCGGATCCTCAGTCATCCACAGATAAAAGGCTATC

The EGFL6 knockout cells were seeded in six-well plates for 24 h in DMEM with 10% FBS before transfection. The cells were transfected with pCDH513b-EGFL6 using the ViaFect Transfection Reagent.

### Cell Proliferation Assay

The cell viability was measured using a cell counting Kit (CCK-8, Dojindo Molecular Technologies Inc., Cat: CK04). The ovarian cancer cells with the EGFL6 gene knockout were transferred into 96-well plates at a density of 2,500 cells/well and incubated overnight. The proliferation rate of the cells was determined by CCK-8 according to the manufacturer's protocol. The optical density values at 450 nm (OD_450_) at the time of 0 h (time point of cell attachment), 24, 48, 72, and 96 h were measured using the microplate reader (Bio-Tek, USA).

### Colony Formation Assay

The ovarian cancer cells with EGFL6 gene knockout were transferred into six-well plates at a density of 1,000 cells/well. After culturing for about 7 days, the cells were fixed with 4% paraformaldehyde for 15 min and stained with 1% crystal violet for 30 mins. The colonies were counted immediately after washing with PBS.

### Wound Healing Assay

The ovarian cancer cells with EGFL6 gene knockout were transferred into six-well plates (3.0 × 10^5^ cells/well) and incubated for 24 h, the cell scratches were made using pipette tips, and the medium was changed to DMEM with 0.5% FBS. After incubation for 48 h, the migrated cells were imaged with the inverted microscope (IX71, Olympus, Japan). The cell migration rate of each group was calculated.

### Migration and Invasion Assays

The ovarian cancer cells with the EGFL6 gene knockout (2 × 10^4^ cells) were seeded in the transwell chamber (8 μm, BD Biosciences) in 200 μl of DMEM basic medium. In the outer chamber, DMEM medium (650 μl) with 10% FBS was used as a chemo-attractant.

The inserts coated with 45 μl of Matrigel (BD Biosciences, Cat: 356234) were used for the assessment of invasion. The inserts uncoated with Matrigel were used for migration. After 24 h, the cells that migrated to the lower surface of the insert membrane were fixed with 4% paraformaldehyde and stained with 0.1% crystal violet (Meryer, Shanghai, China), and imaged using the inverted microscope.

### Tube Formation Assay

The 96-well plates were maintained at 4°C and coated with 60 μl of Matrigel matrix per well. The HUVEC cells (1 × 10^4^ cells/well) suspended in DMEM medium with Low Serum Growth Supplement (Gibco) were transferred to each well. Conditioned medium (100 μl) of the ovarian cancer cells with EGFL6 gene knockout was added into the wells and the plates were incubated for 6 h at 37°C in a 5% CO_2_ incubator. The tube formation was observed under a microscope and the tube numbers were counted in five random high-power fields.

### Apoptosis Assay

Digested using trypsin without EDTA, the ovarian cancer cells with EGFL6 gene knockout were collected via centrifugation and washed with PBS twice. Then, the cells were incubated in Annexin V Binding Solution with Annexin V–FITC and PI (Dojindo Molecular Technologies, AD10-10) for 30 min. The cells were analyzed using a BD Accuri^TM^ C6 Plus.

### Xenograft Model of Ovarian Cancer

All the animals used in the experiments were treated humanely in accordance with Institutional Animal Care and Use Committee guidelines of Jinan University. Female BALB/C nude mice (4–5 weeks) were purchased from Beijing Huafukang Biological Co., Ltd. The BALB/C nude mice were fed in a specific pathogen free (SPF) environment with the temperature maintained at 25–27°C.

In the ovarian tumor xenograft experiments, the ovarian cancer cell lines with EGFL6 gene knockout, E10 and G11 (6 × 10^6^ cells in 100 μl PBS), were injected subcutaneously in the right shoulder of BALB/c nude mice (*n* = 8). Tumor size was measured with a vernier caliper every 3 days. Tumor volume was calculated using the formula *V* = 0.52*ab*^2^ (*a* = tumor length, *b* = tumor width). After 3 weeks, the mice were euthanized and tumors were stripped for further analysis.

### Immunohistochemistry Assays

The experimental procedure for immunohistochemistry was performed as described previously (23). The primary antibodies were rabbit anti-CD31, rabbit anti-LYVE1 polyclonal antibodies (Abcam, 1:50, Cat: ab28364, ab14917) and rabbit anti-human EGFL6, PDGFB polyclonal antibody, rabbit anti-human monoclonal antibodies of VEGFA, and FGF-2 (Abcam, 1:100, Cat: ab140079, ab23914, ab52917, ab92337). The secondary antibody was HRP-conjugated goat anti-rabbit IgG (Servicebio, 1:200, Cat: G1215).

### Transcript Profile Assay

For the mRNA-seq assay, the *EGFL6* knockout ovarian cancer cells (E10 cells) were submitted to Shanghai Majorbio Bio-pharm Technology Corporation for RNA-seq. Poly(A) RNA was purified from the total RNA and then converted to double-stranded cDNA; the resulting cDNA samples were sequenced using the standard Solexa protocols. Gene ontology (GO) enrichment and Kyoto Encyclopedia of Gene and Genomes (KEGG) pathway analyses were performed with DAVID (Database for Annotation, Visualization, and Integrated Discovery). The data were analyzed on the free online platform of Majorbio Cloud Platform, and each step was strictly in accordance with the transcriptome sequencing criteria.

### Statistical Analysis

Statistical analyses were carried out using one-way ANOVA, followed by the least significant difference test using the statistical software GraphPad Prism 6. Data were represented as mean ± SD. *p* values < 0.05 (^*^) and *p* < 0.01 (^**^) were considered statistically significant.

## Results

### Expression of *EGFL6* in Ovarian Cancer Cell Lines and Clinical Samples

**Figure 1:**
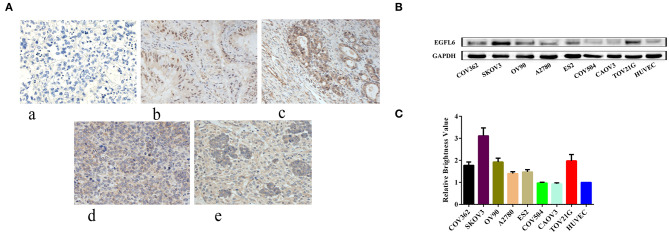
Expression of EGFL6 in different ovarian cancer cells and ovarian cancer tumors from different patients. **(A)** Immunohistochemistry assay of protein expression of EGFL6 in four patient ovarian cancer tissues and one normal ovarian tissue sample. (a) Normal human ovarian tissue, (b) moderately differentiated mucinous adenocarcinoma, (c) ovarian clear cell carcinoma, (d) poorly differentiated malignant tumor, and (e) high-grade serous papillary carcinoma. **(B)** EGFL6 expression levels analyzed by Western blot in different ovarian cancer cells. **(C)** Quantitative analysis of EGFL6 expression in different ovarian cancer cells.

Tumor tissues from four patients with malignant ovarian cancer of different subtypes and one sample of normal ovarian tissue were analyzed. Regardless of the subtypes and differentiation status of the cancers, EGFL6 was overexpressed in all four patients with malignant ovarian cancers (Figure 1A), while the normal ovarian tissue showed minimal EGFL6 expression.

The expression of EGFL6 was detected in a panel of human ovarian cancer cells (CAOV3, COV-362, COV-504, EFO-27, ES-2, OV-90, SKOV3, and TOV-21G), and EGFL6 expression in HUVECs served as a negative control (Figure 1B). The results showed that EGFL6 was expressed in all the ovarian cancer cell lines but with variations. SKOV3 highly expressed EGFL6 and was therefore, selected as a representative for further studies (Figure 1C).

### *EGFL6* Knockout and the Establishment of Single Cell Clones

**Figure 2:**
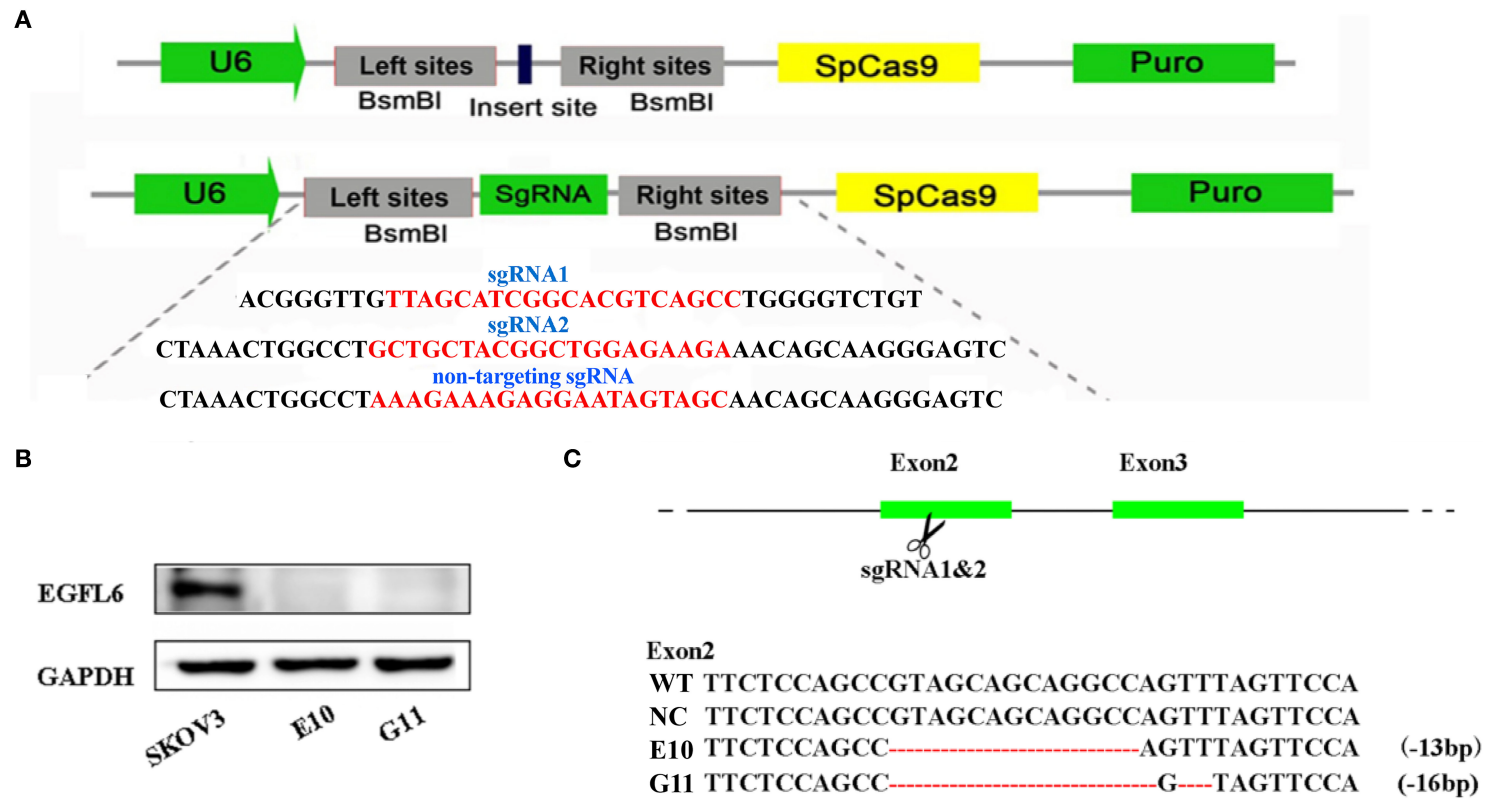
Gene knockout of EGFL6 by CRISPR/Cas9 and identification of SKOV3 cells. The sgRNA was designed according to exon 2 of the human EGFL6 gene. The CRISPR/Cas9 lentivirus was packaged in 293T cells, SKOV3 cells were infected with CRISPR/Cas9 lentivirus, cultured using puromycin, and the monoclonal cell lines were screened using the limited dilution method. **(A)** Construction of sgRNA into lentiCRISPRv2 plasmid. **(B)** Western blot for EGFL6 in E10 and G11, validating the loss of protein expression. **(C)** Sequences of wild-type SKOV3 (WT), negative control group (NC), and the selected clones E10 and G11, validating the deletion of base pairs in exon 2.

The gRNA was designed according to the target sites in exon 2 of human *EGFL6* using an online tool developed by Prof. Zhang (http://crispr.mit.edu/). Specific gRNAs were inserted into lentiCRISPR V2 vectors, as shown in Figure 2A. The CRISPR/Cas9 lentivirus was packaged in 293T cells. The SKOV3 cells were infected with the CRISPR/Cas9 lentivirus and cultured with puromycin; the single cell clones were selected by the limited dilution method. The selected single cell clones were expanded and cultured, and the extracted total protein was used for verification of EGFL6 expression by Western blot. The sub-clones of E10 and G11 no longer expressed EGFL6 protein (Figure 2B). The target gene sequence of *EGFL6* was identified by PCR amplification and sequencing. The sequencing results showed that E10 and G11 clones had deletions of 13 and 16 base pairs (bps) in exon 2, respectively (Figure 2C). The results demonstrated that *EGFL6* was successfully knocked out in the E10 and G11 clones.

### Viability, Apoptosis, and Induction of Tube Formation by *EGFL6* Knockout Ovarian Cancer Cells

**Figure 3:**
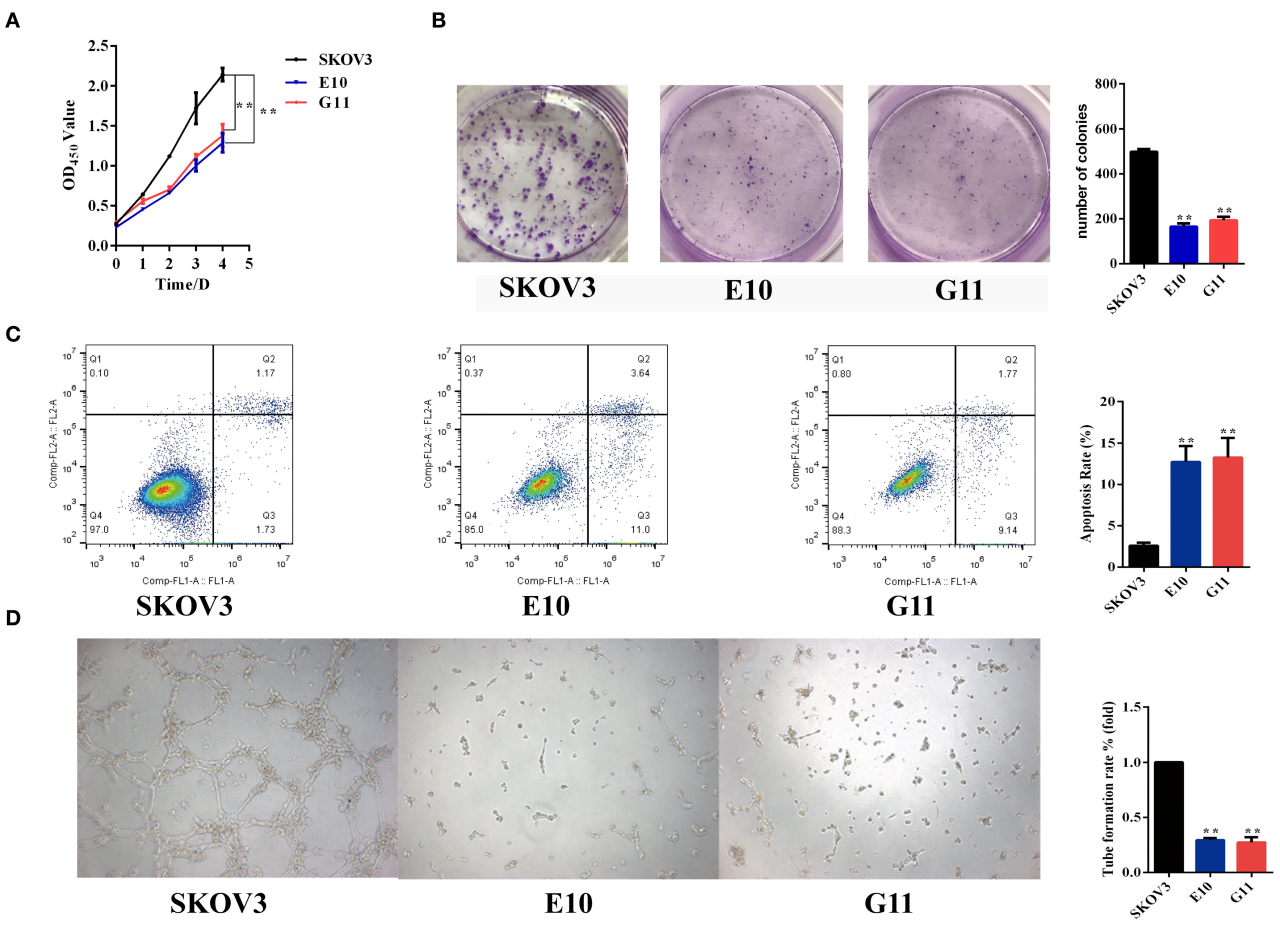
Inhibition of proliferation, angiogenesis, and promotion of apoptosis in ovarian cancer cells with EGFL6 knockout. **(A)** Cell viability by CCK8 assay. The ovarian cancer cells with EGFL6 gene knockout (2,500 cells/well) were transferred into 96-well plates. The cell viability was measured using cell counting Kit of CCK8. **(B)** Colony formation. The ovarian cancer cells with EGFL6 gene knockout (1,000 cells/well) were transferred into six-well plates with a semisolid medium. **(C)** The apoptosis assay of ovarian cancer cells with EGFL6 knockout by flow cytometry. The cells were incubated in Binding Solution with Annexin V–FITC and PI and then assayed with Flow cytometry. **(D)** Tube formation of HUVEC cells stimulated by conditional media of ovarian cancer cells with EGFL6 knockout. The human endothelial cells HUVEC (1 × 10^4^ cells/well) were added in 96-well plates and the conditioned medium (100 μl) of ovarian cancer cells with EGFL6 gene knockout was added. *p < 0.05 and **p < 0.01 were considered statistically significant.

The cell viability of *EGFL6* knockout clones of SKOV3 (E10 and G11) was determined by CCK-8 assay. The number of viable cells was significantly suppressed in the *EGFL6* knockout cells E10 and G11 (Figure 3A). The OD_450_ of *EGFL6* knockout cells (E10 and G11) was significantly lower than that of the SKOV3 control group. The inhibition rate reached 40% (*p* < 0.01) in E10 and 36% (*p* < 0.01) in G11. The colony formation ability of the *EGFL6* knockout cells was examined by the plate colony formation assay. The results showed that the colony-forming ability of the *EGFL6* knockout cells (E10 and G11) was significantly suppressed (Figure 3B).

Apoptosis was examined in the *EGFL6* knockout cells. The results showed that the apoptosis rates of E10 and G11 cells were 12.72% (*p* < 0.01) and 13.25% (*p* < 0.01), respectively (Figure 3C), which were significantly higher than that of the SKOV3 cells. These data indicate that knockout of *EGFL6* could significantly inhibit proliferation and colony formation, as well as promote apoptosis in ovarian cancer cells.

To test the role of EGFL6 in stimulating angiogenesis in ovarian cancer cells, the conditioned media were collected from the cultures of E10, G11, and SKOV3 control group cells. The tube formation ability of HUVEC cells was tested in Matrigel-coated plates with conditioned media stimulation. The conditioned media of E10 and G11 cells showed reduced tube formation rates of 71% (*p* < 0.01) and 72% (*p* < 0.01), respectively, compared to that of SKOV3 cells (Figure 3D). These data showed that *EGFL6* knockout could significantly inhibit tube formation by HUVEC.

### Inhibition of Migration and Invasion of *EGFL6* Knockout Ovarian Cancer Cells

**Figure 4:**
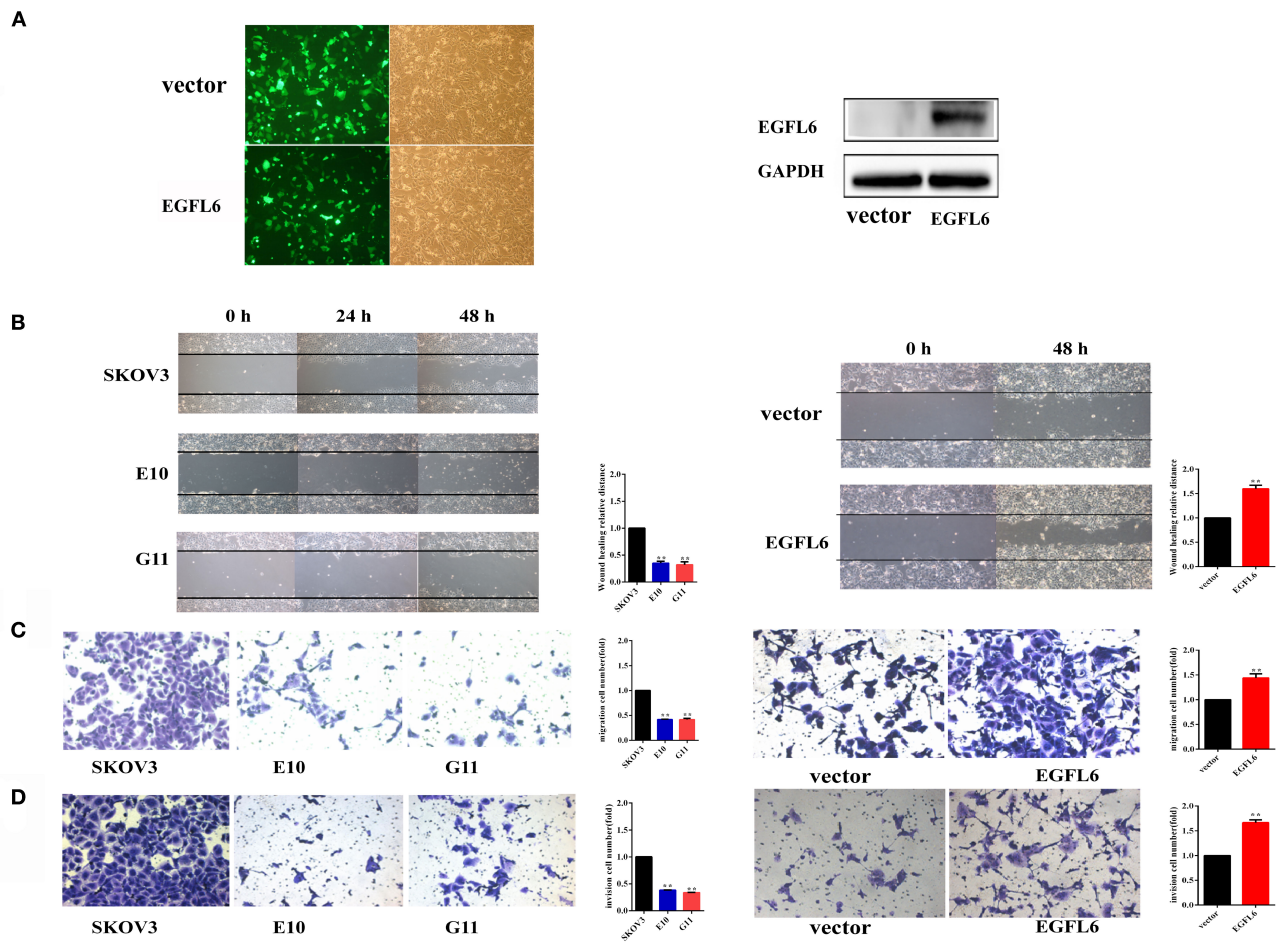
Knockout of EGFL6 gene inhibits migration and invasion of ovarian cancer cells. The cells (2 × 10^4^) of E10, G11, and SKOV3 were seeded in the transwell chamber. The inserts coated with 45 μl of Matrigel matrix were used for the assessment of invasion, and uncoated inserts were used for migration. **(A)** The fluorescence analysis of transfected E10 cells and Western blot analysis of EGFL6 protein expression in E10 cells. **(B)** Migration assay by scratch healing test. **(C)** Migration assay using a transwell. **(D)** Invasion assay using a transwell. *p < 0.05 and **p < 0.01 were considered statistically significant.

The *EGFL6* knockout cells E10 and G11 were cultured in six-well plates (3.0 × 10^5^ cells/well) and incubated for 24 h and the scratches were made. The scratch test showed that the migration distances of the *EGFL6* knockout cells E10 and G11 were significantly shorter than that of the SKOV3 control group cells. The migration rate in E10 and G11 cells were reduced by 65% (*p* < 0.01) and 68% (*p* < 0.01), respectively. The re-expression of EGFL6 in the knockout cells E10 was conducted by transfection with the recombinant pCDH513b-EGFL6 plasmid (Figure 4A). The migration ability of E10 cells was increased by 59% (*p* < 0.01) after re-expression of EGFL6 (Figure 4B).

The migration and invasion of *EGFL6* knockout cells were further evaluated by the Matrigel uncoated and coated transwell assays. In the transwell, the results showed that the migration rate was reduced by 58% (*p* < 0.01) in both E10 and G11 cells compared to that in the SKOV3 control group cells. When EGFL6 was re-expressed in E10, the migration rate was increased by 44% (*p* < 0.01) (Figure 4C). Cell invasion was evaluated in Matrigel-coated transwell. The invasion was reduced by 62% (*p* < 0.01) in E10 cells and 66% (*p* < 0.01) in G11 cells, respectively. Moreover, the re-expression of EGFL6 in the E10 cells increased the invasion rate by 66% (*p* < 0.01) (Figure 4D). These results revealed that the knockout of *EGFL6* significantly inhibited the migration and invasion of ovarian cancer cells.

### Inhibition of Ovarian Tumor Growth and Angiogenesis *in vivo*

**Figure 5:**
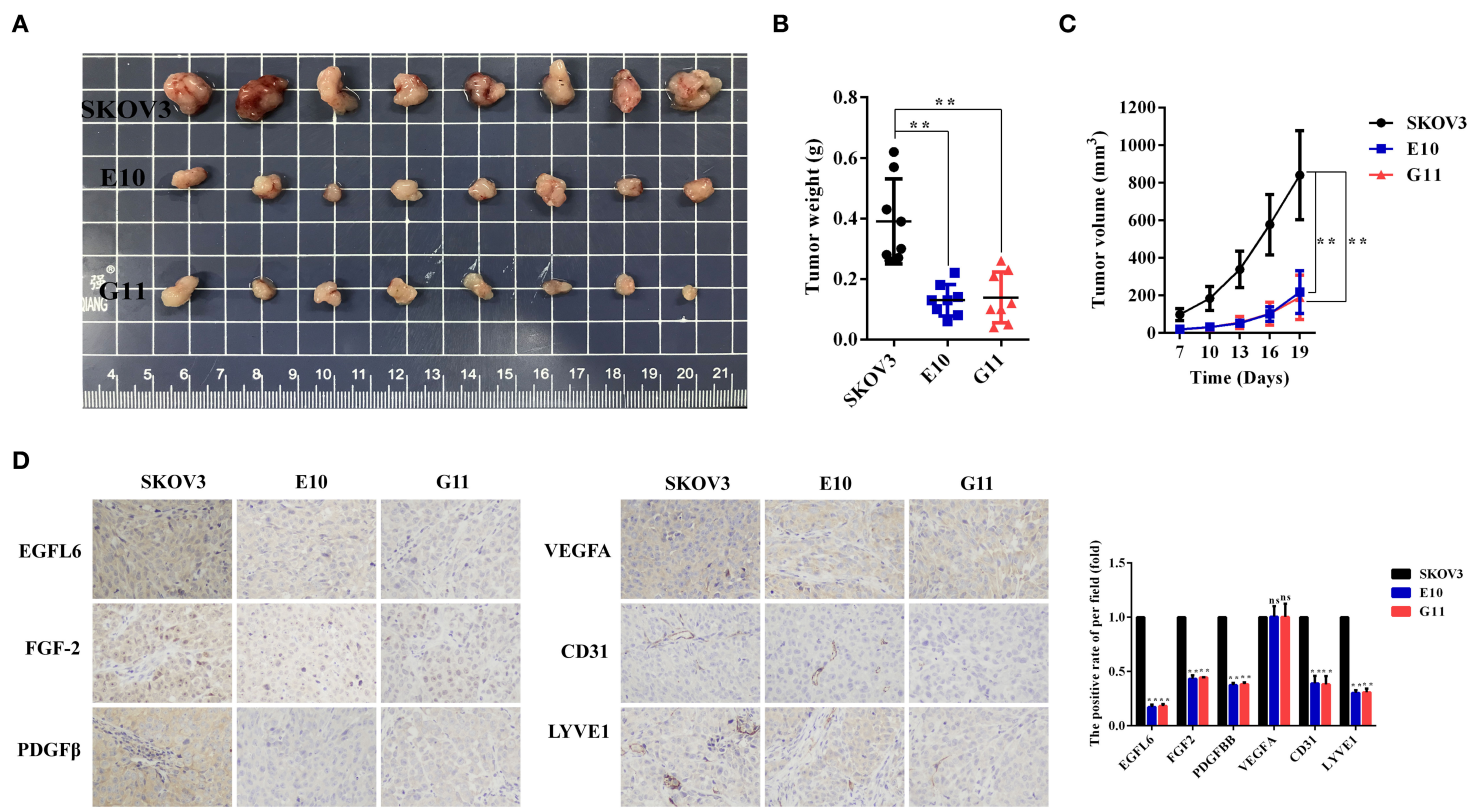
Effect of EGFL6 knockout on tumor growth and angiogenesis in the ovarian cancer nude mouse model. Ovarian cancer cells with EGFL6 knockout (E10 and G11, 6 × 10^6^ cells in 100 μl PBS) were injected subcutaneously in the right shoulder of BALB/c nude mice (*n* = 8). The SKOV3 cells were the control. **(A)** Tumors stripped from the nude mice in different groups. **(B)** The curve of tumor weight changes in the nude mice in different groups. **(C)** Growth curve of tumors in different groups. **(D)** Immunohistochemical assay of tumor blood and lymphatic vasculature and angiogenic factors. The blood vascular network was stained with anti-CD31 antibody. The lymphatic vasculature was stained with anti-LYVE1 antibody. The angiogenic factors were assayed with the antibodies against EGFL6, FGF2, PDGFB, and VEGFA. *p < 0.05 and **p < 0.01 were considered statistically significant.

The SKOV3 control group and the *EGFL6* knockout cells E10, G11 (6 × 10^6^ cells per injection) were subcutaneously injected into BALB/C nude mice for tumor formation. The body weight and tumor volume were monitored and recorded. The mice were sacrificed after 4 weeks and the tumors were stripped, weighed, and photographed (Figure 5A). The results showed that the final tumor weight (Figure 5B) and longitudinal tumor growth (Figure 5C) of both E10 and G11 groups were significantly lower than that of the control group. At the end of the experiment, the mean tumor weight of the E10 and G11 groups were 66 and 64% (*p* < 0.01) lower than that of the control group, respectively. The average tumor volumes of the E10 and G11 groups were 74 and 77% (*p* < 0.01) lower than that of the control group, respectively.

The tumor tissues in the groups E10 and G11 were paraffin-embedded and the sections were immunohistochemically stained with antibodies for EGFL6, FGF-2, PDGFβ, VEGFA, CD31, and LYVE1. The results showed that the expression levels of EGFL6 in E10 and G11 cells were reduced by 83 and 82% (*p* < 0.01), respectively. The microvessel density (CD31) and lymphatic vessel (LYVE1) density in tumor tissues decreased significantly. Additionally, the expression levels of FGF-2 and PDGFβ in E10 as well as G11 tumor tissues were decreased (Figure 5D).

### Transcript Profile Assays of Ovarian Cancer Cells With *EGFL6* Knockout

**Figure 6:**
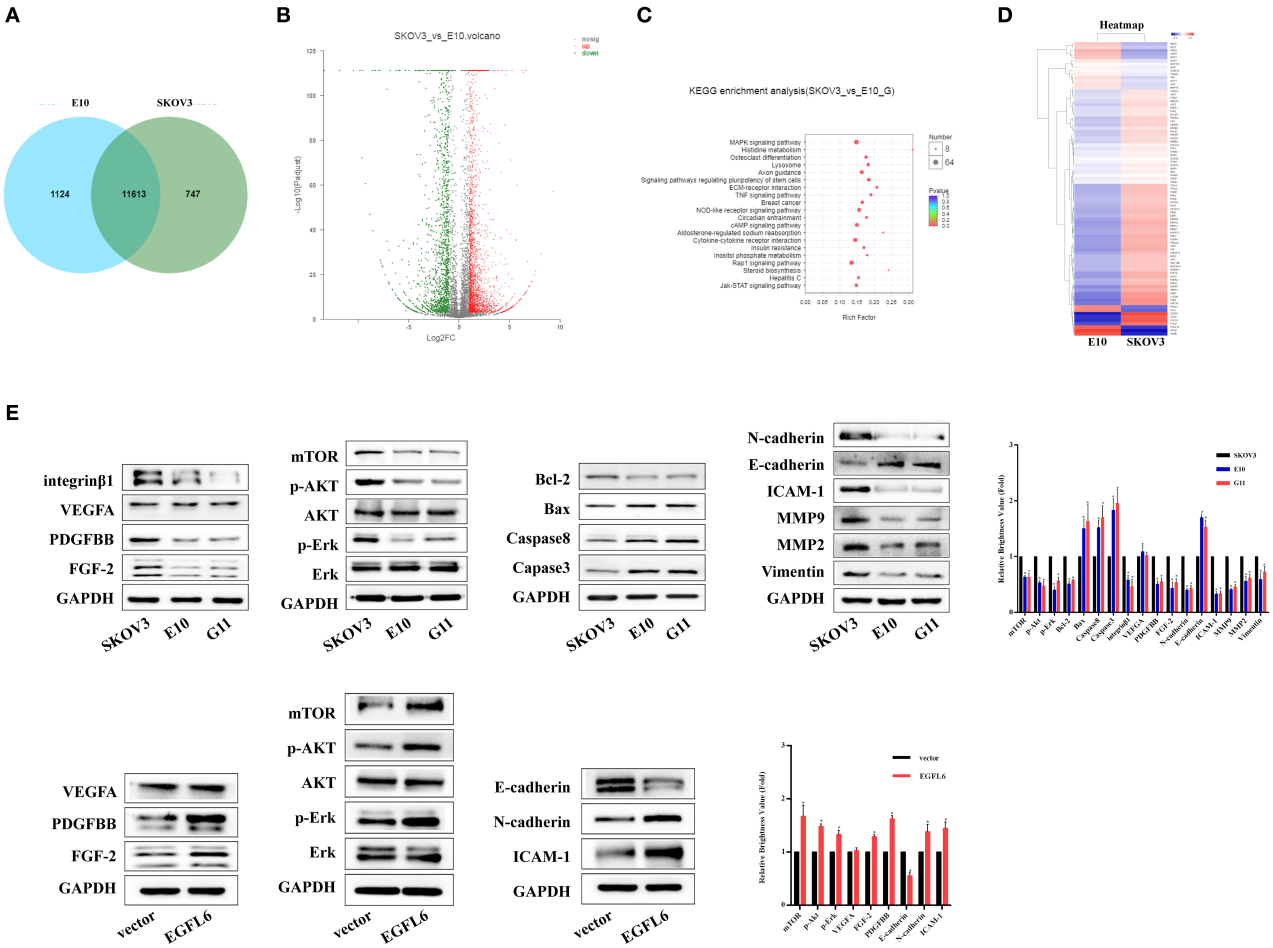
Transcriptome analysis of ovarian cancer cells with EGFL6 knockout. **(A)** Venn diagram shows the number of co-expressed genes and unique genes expressed by SKOV3 and E10, respectively. **(B)** Scatter plot of co-expressed genes between SKOV3 and E10. **(C)** KEGG enrichment of scatter plot. **(D)** Cluster analysis of differential gene expression pattern. **(E)** Western blot assays of signal pathways related to proliferation, apoptosis, migration, invasion, and angiogenesis in the cancer cells with EGFL6 knockout. *p < 0.05 were considered statistically significant.

Transcript profile assays were performed by Shanghai Majorbio Bio-pharm Technology Corporation. A Venn diagram displays the co-expressing genes and the special expression genes in the *EGFL6* knockout cell E10 and SKOV3 control group, as shown in Figure 6A. There were 11,631 genes co-expressed in the knockout cell line E10 and cell line SKOV3, with 1,124 genes expressed only in E10 cells and other 747 genes expressed only in SKOV3 (Figure 6A). The Poisson distribution analysis was used to screen the differentially expressed genes (DEGs) between E10 and SKOV3 cells. The DEGs were used for further functional analysis. The results showed that 4,420 genes were significant differential expression. In the 4,420 DEGs, 2,385 genes were up-regulated and 1,835 genes were down-regulated in the E10 cells (Figure 6B).

The pathway enrichment analysis revealed that all the genes had a Kyoto Encyclopedia of Genes and Genomes (KEGG) annotation and the 4,420 DEGs were assigned to 336 KEGG pathways. The scatter plot for these enriched results was generated (Figure 6). KEGG enrichment analysis showed that knockdown of *EGFL6* could affect MAPK signaling pathway, TNF, ECM, and Jak-STAT signaling pathways in ovarian cancer cells (Figure 6C). These results are consistent with the cell function experiments, which showed that *EGFL6* was associated with proliferation, metastasis, and angiogenesis in ovarian cancer cells.

A total of 87 genes related to proliferation, migration, invasion, and angiogenesis of ovarian cancer cells were selected for clustering heat map assays. The data showed that the expression of FGF2, PDGFB, NOTCH1, and MAPK was significantly downregulated in E10 cells, while the expression of CXCL10, TNF, CASP10, and MMP was significantly upregulated (Figure 6D).

In order to verify the relationship between *EGFL6* downregulation and proliferation, metastasis, and angiogenesis, the related signaling pathways were assayed. The Western blots were used to validate the changes of some related molecules in these pathways. The results showed that the expressions of FGF-2, PDGFBB, and integrinβ1 were significantly decreased, while the expression of VEGFA showed few changes. The expression of intercellular adhesion molecule 1 (ICAM-1) was reduced in E10 and G11 cells, which may also be a stimulating factor for tumor metastasis. On the other hand, knockout of *EGFL6* in ovarian cancer cells could reduce the phosphorylation level of Erk, Akt, and mTOR. In addition, proteins associated with apoptosis, caspase3, caspase8, Bax, and Bcl-2 were altered after knocking out *EGFL6*. Knockout of *EGFL6* could inhibit the expression of N-cadherin, MMP9, MMP2, and vimentin and promote the expression of E-cadherin, which indicated that the knockout of *EGFL6* may result in the inhibition of EMT in ovarian cancer cells. Furthermore, after re-expression of the EGFL6 gene in E10 cells, the expression of these proteins showed corresponding changes (Figure 6E).

## Discussion

The EGFL6 gene is highly expressed in embryos and most tumor tissues (14–16). Buckanovich et al. (27) found that EGFL6 was abundantly expressed in breast cancer, lung cancer, meningioma, melanoma, and ovarian cancers compared with normal tissues, and proposed that EGFL6 was a tumor blood vessel protein and a biological marker of ovarian cancer. The role of EGFL6 in carcinogenesis has been investigated in ovarian cancer but the mechanism is unclear.

Proliferation plays an important role in the carcinogenesis of tumors and is usually accompanied by cell cycle regulation and apoptosis (28). Zhang et al. (29) and An et al. (23) reported that knockdown of *EGFL6* by shRNA could promote the apoptosis of colorectal cancer and breast cancer, causing the change of downstream Caspase-3 and Caspase-9. In our study, we found that *EGFL6* was highly expressed in ovarian cancer tissues from patients, regardless of subtypes of the tumor. Moreover, we found that deletion of EGFL6 could decrease the expression of Bcl-2 and increase the expression of Bax and Caspase-8, which could activate the Caspase-3 signal pathway and cause apoptosis of ovarian cancer cells. However, the mechanism connecting EGFL6 and the cascade signal pathway associated with ovarian cancer cell apoptosis requires further investigation.

Zhang et al. (29) reported that silencing of EGFL6 induced cell cycle arrest with an accumulation of cells in the G0/G1 phase and inhibition of the G1-S transition, consistent with the results reported regarding ovarian cancer through the regulation of the Wnt/β-catenin signaling pathway. In the canonical Wnt signaling pathway, Wnt pathway activation increases the expression of β-catenin which enters the nucleus and engages TCF transcription factors, such as TCF7L2, activating target genes related to Wnt pathway, regulating proliferation, cell cycle and apoptosis.

In addition, some studies have found that EGFL6 could play roles through the SHP2/ERK and integrin/Tie2/Akt pathway (22, 23). The other studies have indicated that EGFL6 may bind to integrin proteins through RGD domain interaction with integrin (30, 31). In this study, we found that the knockout of EGFL6 could affect the signaling pathways for ovarian cancer tumorigenesis, by activation of the integrin/Akt/mTOR and MAPK/Erk pathways.

The MAPK and Akt signaling cascade are downstream signal pathways for growth factor–receptor interactions. The PI3K/Akt/mTOR and Ras/MAPK signaling pathways could be activated by a variety of physiological stimuli, including EGF, IGF, and FGF (32). Furthermore, Kano et al. (33) has reported that VEGFA and FGF-2 synergistically could promote neovascularization by enhancing endogenous PDGFB–PDGFRβ neovascularization. It has been demonstrated that FGF-2 could promote the expression of PDGFB mRNA (34, 35). In this study, we found that the expression of FGF-2, PDGFB related to tumor angiogenesis was significantly decreased after knocking out EGFL6 *in vivo* and *vitro*. Furthermore, the results of heat map analysis showed that the expression of FGF-2, FGFR-2, PDGFB, PDGFR, and other growth factors were downregulated after the knockout of EGFL6. It was related with the PI3K/Akt pathway transmits proliferation and anti-apoptotic survival signals in cancer (36–38). Besides, activation of Ras/ERK pathway has been regarded as one of the most important events in growth factor signaling. Therefore, it is clear that there is crosstalk between the PI3K/Akt and Ras/ERK pathways via the direct interaction of Ras and PI3K (39, 40). In conclusion, this suggests that promotion of the PI3K/Akt signaling pathway plays a role in the tumorigenesis of ovarian cancer via promotion of cell proliferation and inhibition of apoptosis.

We also found that EGFL6 could promote metastasis through the EMT signal pathway in ovarian cancer tumor. As a potential ligand of EGFR, EGFL6 could downregulate the expression of E-cadherin, upregulate the expression of F-actin and Vimentin, and promote metastasis of nasopharyngeal carcinoma through the EMT signal pathway (41). Our results indicated that the knockout of the EGFL6 gene could inhibit EMT in ovarian cancer cells by upregulating the expression of E-cadherin and downregulating the expression of N-cadherin, MMP2, MMP9, and Vimentin. EMT could be regulated by a variety of factors, such as TGFβ, FGF-2, PDGFB, VEGF, and other growth factors, which play an important role in its induction of EMT (42, 43). These growth factors activate Smad, MAPK, PI3K/Akt, Wnt, Notch, and other signaling pathways through the corresponding receptors, and upregulate and activate Snail, ZEB1, ZEB2, and other EMT-related transcription factors through a signal cascade reaction. These transcription factors inhibit epithelial marker molecules, upregulate interstitial marker molecules, and cause EMT of ovarian cancer cell (44, 45).

**Figure 7:**
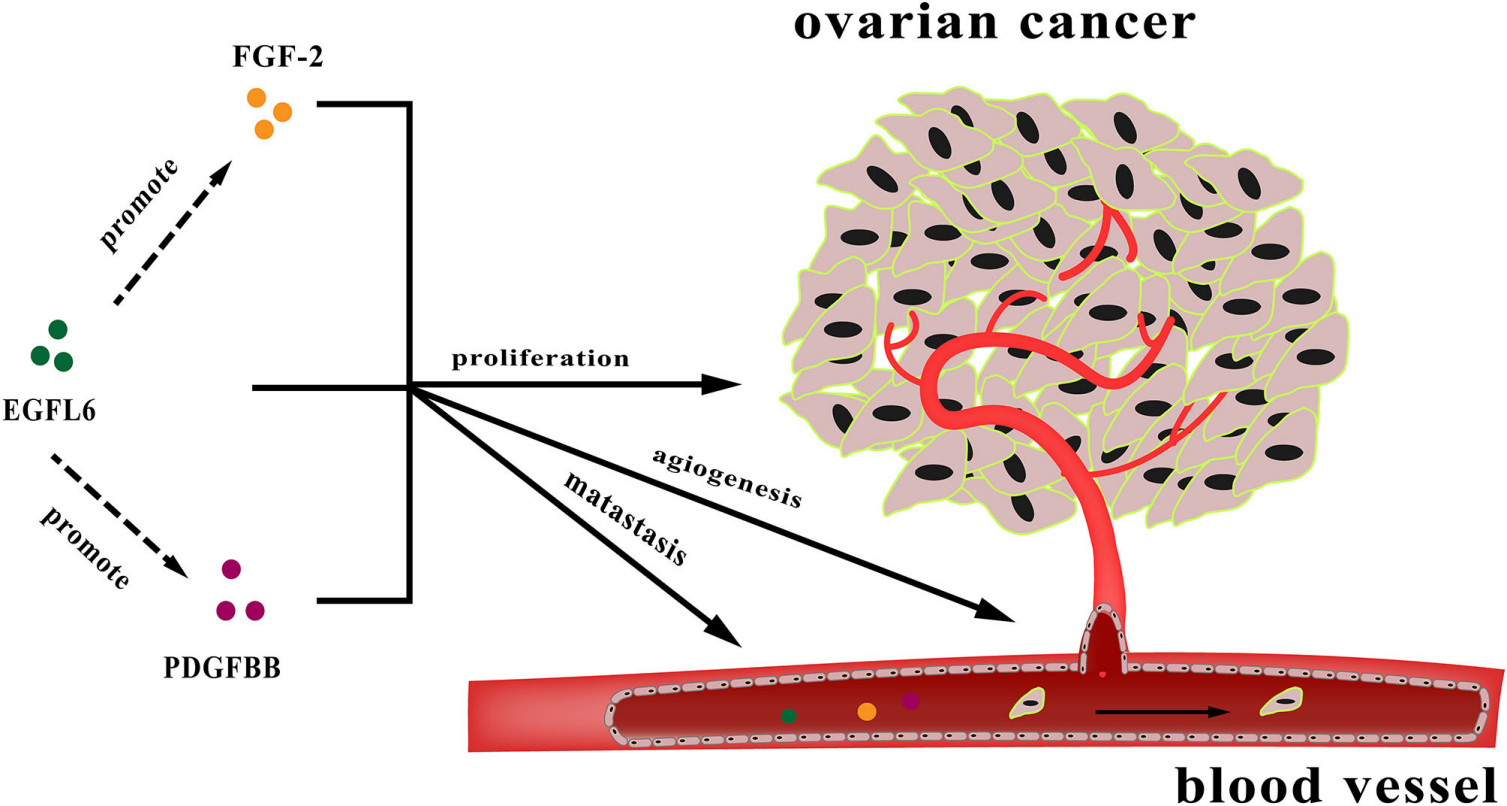
The diagram of regulation by EGFL6 in tumor angiogenesis.

In conclusion, EGFL6 is an oncogenic gene involved in multiple pathways influencing ovarian cancer cell proliferation, metastasis, and angiogenesis. These functions are partially regulated by the downstream genes FGF-2 and PDGFB (Figure 7). However, the specific regulatory mechanism is still unclear and needs to be further studied. EGFL6 might serve as a therapeutic target and prognostic marker for ovarian cancer. Therefore, understanding the function of EGFL6 in ovarian cancer could have potential implications for diagnosis and therapy.

## Data Availability Statement

The datasets presented in this study can be found in online repositories. The names of the repository/repositories and accession number(s) can be found below: the NCBI sequence read archive (SRA): SRR12147538; SRR12147539.

## Ethics Statement

The animal study was reviewed and approved by Laboratory Animal Ethics Committee of Jinan University.

## Author Contributions

WZ, TL, and ND designed the project. WZ, TL, and SZ designed the experiments. WZ, CL, YZ, and SZ performed the experimental work. WZ, CL, and YZ analyzed the results. WZ, CL, QC, and ND wrote the manuscript. All authors contributed to the article and approved the submitted version.

## Conflict of Interest

The authors declare that the research was conducted in the absence of any commercial or financial relationships that could be construed as a potential conflict of interest.
